# Mitogenomic variation across cultivated and wild beet (*Beta vulgaris* L.) accessions reveals patterns associated with cytoplasmic male sterility

**DOI:** 10.1186/s12870-026-09766-2

**Published:** 2026-08-17

**Authors:** Weiting Zhong, Shuo Zhang, Junhu Kan, Yanghe Zhao, Zhiqiang Wu, Luke R. Tembrock, Dali Liu, Xiaolin Gu, Wang Xing

**Affiliations:** 1 National Beet Medium-Term Gene Bank, Heilongjiang University, Harbin, 150080 China; 2 Shenzhen Branch, Guangdong Laboratory of Lingnan Modern Agriculture, State Key Laboratory of Tropical Crop Breeding, Key Laboratory of Synthetic Biology, Ministry of Agriculture and Rural Affairs, Agricultural Genomics Institute at Shenzhen, Chinese Academy of Agricultural Sciences, Shenzhen, China; 3 College of Plant Science & Technology, Huazhong Agricultural University, Wuhan, 430070 China; 4 School of Medical, Molecular and Forensic Sciences, Murdoch University, Murdoch, WA 6149 Australia; 5 Department of Agricultural Biology, Colorado State University, Fort Collins, CO 80523 USA; 6 China National Seed Group Co., Ltd., Sanya, 572000 China

**Keywords:** Sugar beet, Mitochondrial genome, CMS, Molecular evolution, Intracellular transfer

## Abstract

**Background:**

Beet hybrid production depends on cytoplasmic male sterility (CMS), which is determined by the mitochondrial genome. Systematically comparing mitochondrial genome structure, evolutionary dynamics, and inter-organellar DNA transfer across cultivated beet types and their wild progenitor, is key to understanding the evolutionary basis of CMS-associated features.

**Results:**

We assembled complete mitochondrial genomes for 19 beet accessions, including four cultivated types (sugar, table, fodder, leaf) and one wild beet. Genome sizes ranged from 344 to 381 kb, with wild beet having the largest genome size and highest repeat content. All accessions shared 53 conserved core genes. The CMS‑specific sequence *preSatp6* was present only in sterile lines and absent in wild beet. CMS lines exhibited accelerated evolutionary rates (both synonymous and non‑synonymous substitutions), whereas wild beet showed the lowest rates. We identified 809 plastid‑to‑mitochondrion transfers (PTMTs) and abundant nuclear organellar insertions (NUMTs/NUPTs); both wild beet and CMS lines accumulated more NUMTs compared to other cultivated types. Phylogenetic analysis placed wild beet at the basal position, supporting its ancestral position, and eight accessions (including four CMS lines and four non‑CMS sugar beet accessions) formed a tight, highly supported clade, with *preSatp6* present exclusively in the CMS lines.

**Conclusions:**

Beet mitochondrial genomes combine functional conservation with structural dynamism. Wild beet represents an ancestral state with a larger, repeat-rich genome and slow evolution, while CMS lines show distinctive structural configurations, accelerated evolution, and active cytonuclear interaction. This study provides a genomic framework for understanding CMS evolution and guiding for future breeding.

**Supplementary Information:**

The online version contains supplementary material available at https://doi.org/10.1186/s12870-026-09766-2.

## Introduction

Plant mitochondrial genomes are notably dynamic over evolutionary timescales. They exhibit extensive variation in size, structure, and mutation rate, largely due to frequent homologous recombination mediated by repetitive sequences [1–3]. Such genomic plasticity can lead to cytoplasmic male sterility (CMS), a trait widely exploited in hybrid breeding, which often arises from chimeric genes generated by recombination between repetitive elements and conserved mitochondrial functional genes [4, 5].

Sugar beet (*Beta vulgaris* var. *altissima*) is the world's second most important sugar crop, and its hybrid seed production relies heavily on CMS [6]. Consequently, mitochondrial genome structure and variation directly affect male fertility. Beet mitochondrial genome is characterized by a large size and complex repeats that drive frequent homologous recombination, resulting in extensive rearrangements and structural polymorphisms [7]. Early studies revealed that repeat‑mediated recombination underlies structural variation in Owen-type CMS [8], and three major sterile cytoplasms (Owen, E, and G types) have been identified in sugar beet [9–11]. Among these, the Owen type is by far the most extensively used in commercial hybrid breeding because of its stable sterility and favourable combining ability [6, 9]. In Owen-type CMS, a 387-amino-acid open reading frame (ORF) located upstream of *atp6* is co-transcribed with the core region to produce preSATP6. This protein accumulates on the mitochondrial membrane of sterile lines and forms a specific 200 kDa homomeric complex that is absent in the mitochondria of fertile lines [12]. Given its central role in sugar beet production, understanding the evolutionary and structural features of the Owen-type mitochondrial genome is critical for the effective use and long-term management of CMS germplasm. Despite these insights, several obstacles hinder the application of mitochondrial genomics in breeding. Frequent rearrangements can destabilize CMS-associated markers, and the relationship between mitochondrial stability and sterility remains unclear [13]. Introgression of wild germplasm may induce fertility disorders due to nucleo-cytoplasmic incompatibility [14]. Moreover, the complexity and high recombination activity of the mitochondrial genome complicate the validation of chimeric ORF functions and the engineering of sterile lines through traditional genetic approaches.

Beyond sugar beet, cultivated beets include three other types—table beet (*Beta vulgaris* var. *rapacea*), fodder beet (*Beta vulgaris* var. *lutea*), and leaf beet (*Beta vulgaris* var. *cicla*), each developed under distinct breeding objectives [6]. This diversity provides a unique opportunity to compare how different selection pressures shape mitochondrial genome evolution. Wild beet (*Beta vulgaris* subsp. *maritima*), the progenitor of cultivated beets, retains high mitochondrial genome diversity and lacks CMS-associated sequences in some populations [15, 16], making it an essential reference for tracing domestication history. However, systematic comparisons of mitochondrial genome structural variation, evolutionary dynamics, and CMS association across these types remain limited. To address this, we assembled complete mitochondrial genomes for 19 accessions covering all major cultivated types (including four Owen-type CMS lines and one maintainer line of sugar beet) and one wild accession. We systematically analyzed repeat dynamics, inter-organellar DNA transfer, nucleotide substitution rates, and phylogenetic relationships. Our goals were to elucidate how mitochondrial genomes vary across cultivation types and between CMS and fertile cytoplasms, and to provide a genomic foundation for understanding CMS evolution in beet.

## Materials and methods

### Plant materials and accession codes

A detailed description of all 19 accessions, including their cultivated type and cytoplasmic status, is provided in Supplementary Table S1. The four CMS lines (12‑cms, 14‑cms, 104‑cms, xw‑cms) are confirmed Owen‑type sterile lines based on their breeding records and documented pedigree history. The accession 12‑o is a known maintainer line for the 12‑cms cytoplasm. For the other sugar beet accessions (Tang2, TC10, TC45, TC52, TC54, TC58, TC73), as well as the table, fodder, leaf, and wild beet accessions, fertility phenotypes were not explicitly determined in this study. These accessions are therefore classified as 'non‑CMS' based on the absence of the *preSatp6* sequence (as identified in the Results section), rather than on direct fertility observations.

### Sample collection and sequencing

A total of 19 beet (*Beta vulgaris* L.) accessions were analyzed, including 12 sugar beet accessions (four cytoplasmic male sterile lines: 12-cms, 14-cms, 104-cms, xw-cms; one maintainer line: 12-o; seven additional lines: Tang2, TC10, TC45, TC52, TC54, TC58, TC73), two table beet accessions (HTC, Shi3), two fodder beet accessions (Si3, TC80), two leaf beet accessions (JT01, ZT01), and one wild beet accession (Ye1). All germplasm resources were provided by the National Beet Medium-term Gene Bank at Heilongjiang University (Harbin, China).

Single‑molecule long‑read sequencing was performed on the PacBio Sequel II platform using the HiFi/CCS (circular consensus sequencing) mode, with sequencing libraries prepared from total genomic DNA (without mitochondrial enrichment). Target sequencing depth was determined based on genome size estimates derived from a preliminary genome survey, ensuring sufficient coverage for de novo assembly of mitochondrial genomes.

### Mitochondrial genome assembly and annotation

De novo assembly of mitochondrial genomes was conducted using PMAT v2.1.5 [17] and Oatk [18]. The initial assembly graphs in Graphical Fragment Assembly (GFA) format were visualized and simplified using Bandage v0.8.1 [19] to evaluate the structural integrity and complexity of assembled contigs. To remove nuclear and plastid contaminating sequences, preliminary assemblies were aligned to known mitochondrial reference sequences (NCBI accession numbers NC_002511 and BA000024.1) using BLAST v2.17.0 [20]. Only sequences with high homology to the reference mitochondrial genome were retained.

Mitochondrial genome annotation was performed using the PMGA web server (http://www.1kmpg.cn/pmg/) with the “29 mitochondrial genomes” database as the reference. BLASTN homology searches were used for cross‑validation. Transfer RNA (tRNA) genes were predicted using tRNAscan-SE v2.0 (http://lowelab.ucsc.edu/tRNAscan-SE/). Ribosomal RNA (rRNA) genes were identified by BLAST v2.17.0 [20] searches against known mitochondrial rRNA sequences. The Owen-type CMS-associated sequence *preSatp6* [12] was identified by BLASTN searches against the assembled mitochondrial contigs using the known preSatp6 sequence (GenBank accession AB055270.1) as query, with parameters: word size = 7, e-value = 1e-5, and sequence identity ≥ 90%. Functional annotation of the *preSatp6* ORF was performed using ORFfinder (https://www.ncbi.nlm.nih.gov/orffinder/) to predict the open reading frame and translated protein sequence. Transmembrane domain prediction was carried out using TMHMM v2.0, and protein secondary structure was predicted using PSIPRED v4.0. The presence of *preSatp6* was further validated by mapping PacBio reads back to the identified region to confirm sequence continuity and coverage. Manual curation of rRNA and protein‑coding genes (PCGs) was carried out in Geneious Prime v2022.2.2, with special attention to trans‑splicing genes and verification of start/stop codons. Circular maps of mitochondrial genomes were generated using OGDRAW v1.3.1 [21] (https://chlorobox.mpimp-golm.mpg.de/OGDraw.html).

### Repeat sequence characterization

Self‑sequence alignments were performed across the 19 beet mitochondrial genomes to identify repeats using BLAST v2.17.0 [20] with parameters: word size = 7, e‑value = 1e‑5, minimum length = 30 bp, and sequence identity ≥ 80%. Repeats were classified into four length categories: short (30–100 bp), medium (101–200 bp), long (201–500 bp and 501–1000 bp), and extra‑long (> 1000 bp). The total number and total length of repeats were quantified. To avoid double‑counting, short repeats embedded within longer ones were excluded from total length calculations. Simple sequence repeats (SSRs) were identified using the MISA web tool [22] with thresholds: mononucleotides ≥ 10, dinucleotides ≥ 8, trinucleotides ≥ 5, tetranucleotides ≥ 4, pentanucleotides ≥ 3, and hexanucleotides ≥ 3. All repeat and SSR results were visualized using the ggplot2 v3.3.6 package in RStudio [23].

### Intracellular transferred fragment identification

PTMTs, nuclear plastid DNA fragments (NUPTs), and nuclear mitochondrial DNA fragments (NUMTs) were identified using BLAST v2.17.0 with a word size of 7 and an e‑value threshold of 1e‑5. For PTMT identification, each mitochondrial genome was compared against the plastid genome of the same accession, which was assembled from the same PacBio dataset using PMAT. For NUPT and NUMT identification, the published beet nuclear genome EL10 [24] was used as a common reference for all accessions. This approach allowed identification of nuclear insertions derived from organellar donors, though it only captures insertions relative to the EL10 [24] reference and may not fully reflect accession‑specific nuclear diversity. To improve detection accuracy, only fragments with a continuous alignment length > 100 bp and sequence identity ≥ 80% were retained. Based on sequence similarity between nuclear insertions and their corresponding organellar donors (used to infer relative insertion time), insertions were classified as ancient (similarity < 90%) or recent (similarity > 90%). This threshold is widely used in plant organellar insertion studies to distinguish recently transferred fragments with limited post‑insertional mutation from older insertions that have accumulated substantial nucleotide substitutions over evolutionary time [25, 26]. The 90% cut‑off aligns with the expected substitution rates of plant nuclear and organellar DNAs, where insertions older than approximately 1–2 million years typically fall below this threshold [27]. NUMTs and NUPTs were further categorized into six length classes: Type I (100–200 bp), Type II (201–300 bp), Type III (301–500 bp), Type IV (501–1000 bp), Type V (1,001–2000 bp), and Type VI (> 2000 bp). Results were visualized using ggplot2 v3.3.6 in RStudio.

### Phylogenetic analysis

Phylogenetic trees were constructed separately from mitochondrial and plastid genome sequences. For the mitochondrial genome, shared coding DNA sequences (CDS) from 19 beet mitochondrial genomes were used, with *Chenopodium quinoa* and *Amaranthus tricolor* as outgroups. Protein‑coding genes were extracted using Phylosuite [28]. Multiple sequence alignments were generated using MAFFT [29] (http://mafft.cbrc.jp/alignment/server/large.html), and poorly aligned regions were removed using the GBLOCKS function in Phylosuite [28]. The filtered alignments were concatenated to produce a mitochondrial gene supermatrix.

For the plastid genome, shared conserved genes were aligned and trimmed using GBlocks v0.91b before concatenation. Maximum likelihood (ML) trees were inferred using IQ‑TREE v1.6.12 [30] with 1000 non‑parametric bootstrap replicates. The general time‑reversible (GTR) model with gamma‑distributed rate heterogeneity (+ G) and a proportion of invariant sites (+ I) was selected as the best‑fit model by the built‑in function in IQ‑TREE. All ML trees were visualized and edited using FigTree v1.4.4 (https://tree.b.ed.ac.uk/software/figtree/).

### Intraspecific evolutionary rate estimation

Synonymous (*dS*) and non‑synonymous (*dN*) substitution rates were estimated for concatenated mitochondrial and plastid datasets, including outgroups. The phylogenetic topology inferred from the plastid PCG supermatrix was used as a fixed constraint to ensure comparability between mitochondrial and plastid rate estimates.

Tree files were modified to remove branch lengths and rooting information, retaining only topological structure. Substitution rates were calculated using the codeml program in the PAML v4.9j package. A branch‑specific model (model = 1) was applied, allowing ω (*dN*/*dS*) to vary among branches. Key codeml parameters were set as follows: seqtype = 1 (codon data), CodonFreq = 2 (F3 × 4 model), and NSsites = 0 (uniform ω across sites within a branch).

Cumulative root‑to‑tip branch lengths for *dS* and *dN* were extracted from codeml outputs, representing the total numbers of synonymous and non‑synonymous substitutions from the root to each terminal lineage. These values were mapped onto the corresponding phylogenetic trees in FigTree v1.4.4. Mean root‑to‑tip distances were computed using the castor v1.7.11 package in R.

## Results

### Mitochondrial genome characteristics of beet accessions

We assembled complete mitochondrial genomes for 19 beet accessions, including 12 sugar beets (four sterile lines and one maintainer line), two table beets, two fodder beets, two leaf beets, and one wild beet. The assembled mitochondrial genomes of cultivated beets ranged from 344,560 to 372,169 bp, with mean sequencing depths ranging from 108.6 × to 1,791.0 ×. The wild beet mitochondrial genome was 380,704 bp with a mean read depth of 1,271.1 ×. GC content ranged from 43.7% to 44.0% across all accessions (Table 1; Supplementary Fig. S1). CMS lines showed a uniform genome size (365,891 bp), whereas non-CMS sugar beet accessions exhibited greater variation (344,560–365,894 bp) (Table 1). While genome size and GC content showed limited variation, sequencing depths varied substantially between cultivated beets and the wild accession.

**Table 1:** Metrics of mitochondrial genome assemblies in beet

Sample	Number of contigs	Genome size (bp)	Max. contig length (bp)	Min. contig length (bp)	Mean depth	Maximum depth	Minimum depth	GC (%)	Cultivated type	Fertility/cytoplasm status
12-cms	3	365,891	221,477	48,681	108.6 ×	113.0 ×	88.1 ×	43.7	sugar beet	CMS
14-cms	3	365,891	221,477	56,410	234.5 ×	254.7 ×	231.5 ×	43.7	sugar beet	CMS
104-cms	3	365,891	221,477	56,401	132.2 ×	144.8 ×	101.5 ×	43.7	sugar beet	CMS
xw-cms	3	365,891	221,477	48,681	119.8 ×	133.7 ×	98.7 ×	43.7	sugar beet	CMS
12-o	3	345,008	161,017	74,673	134.8 ×	141.6 ×	127.0 ×	44.0	sugar beet	maintainer (known fertile)
Tang2	3	365,891	221,477	48,681	791.0 ×	831.1 ×	653.2 ×	43.7	sugar beet	non-CMS (fertility not determined)
TC10	3	365,892	221,478	56,410	153.6 ×	159.9 ×	127.6 ×	43.7	sugar beet	non-CMS (fertility not determined)
TC45	3	365,894	221,478	48,681	250.3 ×	266.4 ×	200.8 ×	43.7	sugar beet	non-CMS (fertility not determined)
TC52	3	345,008	161,017	74,673	711.6 ×	739.4 ×	679.0 ×	44.0	sugar beet	non-CMS (fertility not determined)
TC54	3	345,008	147,738	74,673	309.5 ×	325.9 ×	281.0 ×	44.0	sugar beet	non-CMS (fertility not determined)
TC58	3	345,008	161,017	74,673	245.2 ×	263.4 ×	228.0 ×	44.0	sugar beet	non-CMS (fertility not determined)
TC73	3	365,891	221,477	48,681	178.8 ×	186.4 ×	161.0 ×	43.7	sugar beet	non-CMS (fertility not determined)
HTC	3	345,008	245,359	24,976	132.0 ×	184.8 ×	127.0 ×	44.0	table beet	non-CMS (fertility not determined)
Shi3	3	345,009	245,360	24,976	232.8 ×	338.7 ×	217.0 ×	44.0	table beet	non-CMS (fertility not determined)
Ye1	3	380,704	133,290	123,306	1271.1 ×	1346.9 ×	1177.9 ×	43.9	wild beet	wild (fertility not determined)
Si3	3	345,008	245,359	24,976	216.9 ×	311.6 ×	207.0 ×	44.0	fodder beet	non-CMS (fertility not determined)
TC80	3	345,008	147,738	74,673	138.2 ×	159.0 ×	124.7 ×	44.0	fodder beet	non-CMS (fertility not determined)
JT01	3	344,560	147,514	74,673	243.1 ×	260.9 ×	230.0 ×	44.0	leaf beet	non-CMS (fertility not determined)
ZT01	3	372,169	216,782	17	522.1 ×	626.0 ×	527.5 ×	44.0	leaf beet	non-CMS (fertility not determined)

**Fig. 1:**
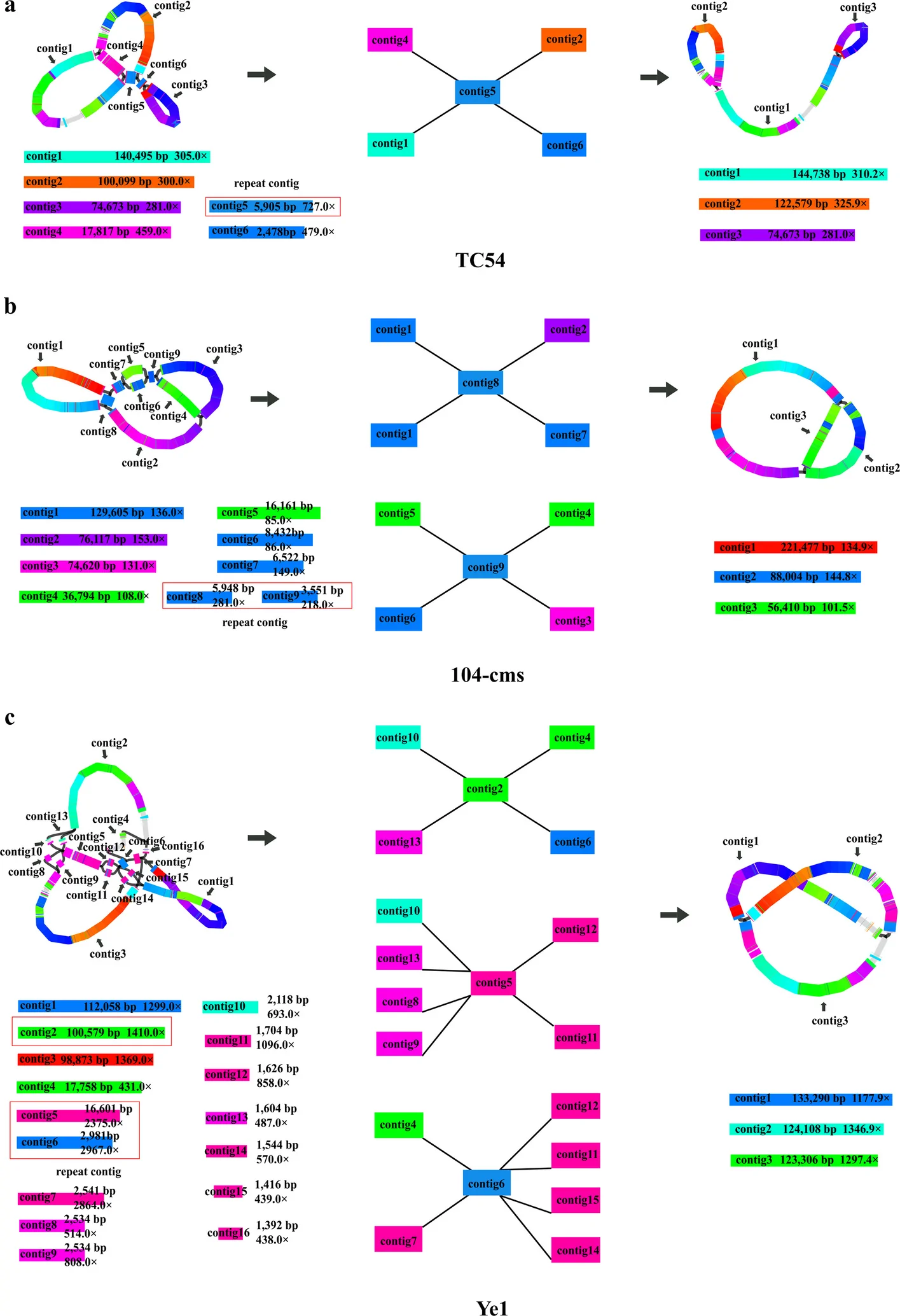
Assembly maps of representative beet mitochondrial genomes showing three structural types based on repetitive contig configuration. **a** Configuration with one repetitive contig (maintainer line 12-o). **b** Configuration with two repetitive contigs (CMS line 12-cms). **c** Configuration with three repetitive contigs (wild beet Ye1). In each panel, assembled contigs are represented as connected nodes; different colours indicate sequence homology between assembled fragments and the reference mitochondrial genome (NC_002511), with identical colours denoting regions of sequence homology. Repetitive sequences are represented as arcs connecting homologous regions. The number of such arcs corresponds to the number of repetitive contigs identified in each genome. The panels illustrate the structural simplification from three (wild/maintainer) to two (CMS) contigs

All mitochondrial genomes contained repetitive contigs. Based on the number of such contigs, the accessions fell into three groups: those with one (Fig. 1a), two (Fig. 1b), or three (Fig. 1c) repetitive contigs. All sterile lines belonged to the two-contig group. Within each group, genome configurations were highly similar. To infer alternative subgenomic conformations, we adopted a graph‑based approach. For each repetitive sequence identified as a recombination mediator, we considered two possible orientations (direct and inverted repeats) that could mediate recombination between flanking regions. By systematically permuting all possible combinations of alternative pathways for each repeat, we generated a set of theoretical conformational variants. These variants were then compared to the observed assembly graph (visualized in Bandage) to identify the most parsimonious set of conformations consistent with the read depth and graph topology. Validation was performed by checking the consistency of junction reads (i.e., reads spanning the repeat boundaries) for each predicted conformation (Supplementary Fig. S2).

Maximum and minimum contig lengths refer to the longest and shortest assembled contigs in each mitochondrial genome assembly, respectively.

### Annotation and identification of potential CMS-associated genes in beet mitochondrial genomes

**Fig. 2:**
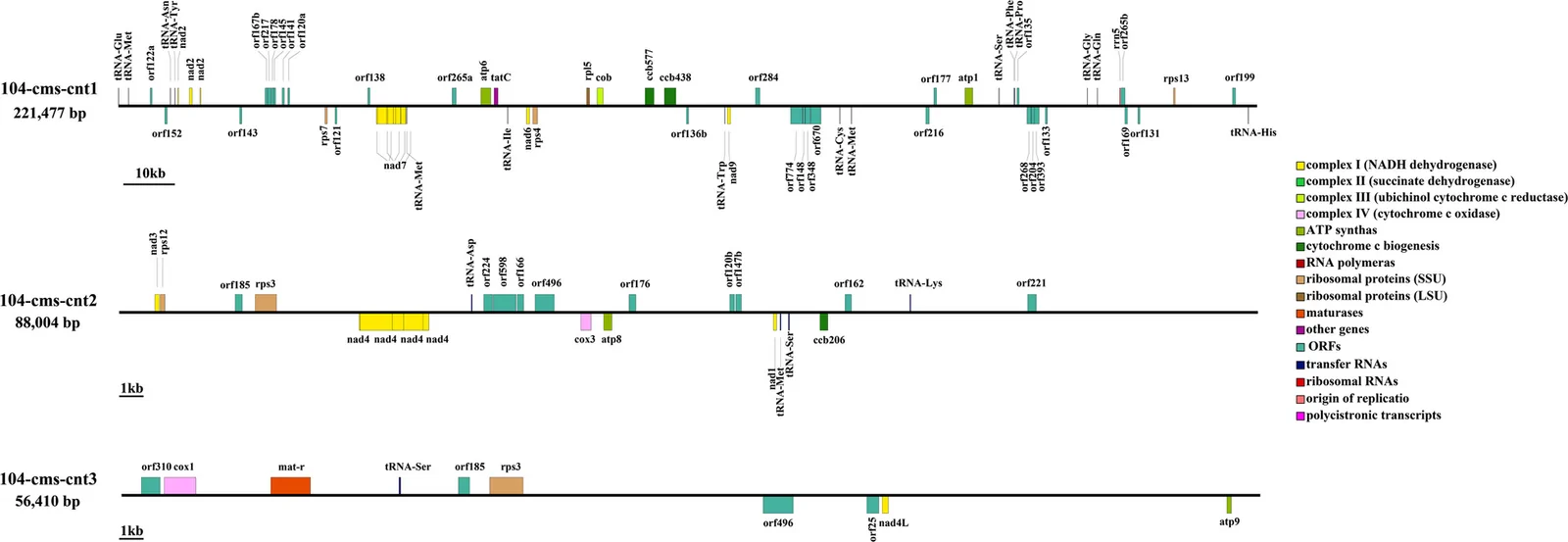
Gene annotation map of three mitochondrial contigs from sugar beet cytoplasmic male sterile line 104-cms. The three contigs (cnt 1, cnt 2, cnt 3) are 221,477 bp, 88,004 bp and 56,410 bp in length, respectively. Genes of different functional categories are labeled with distinct colors, and genes above/below the contig lines indicate forward/reverse transcription directions, respectively

**Fig. 3:**
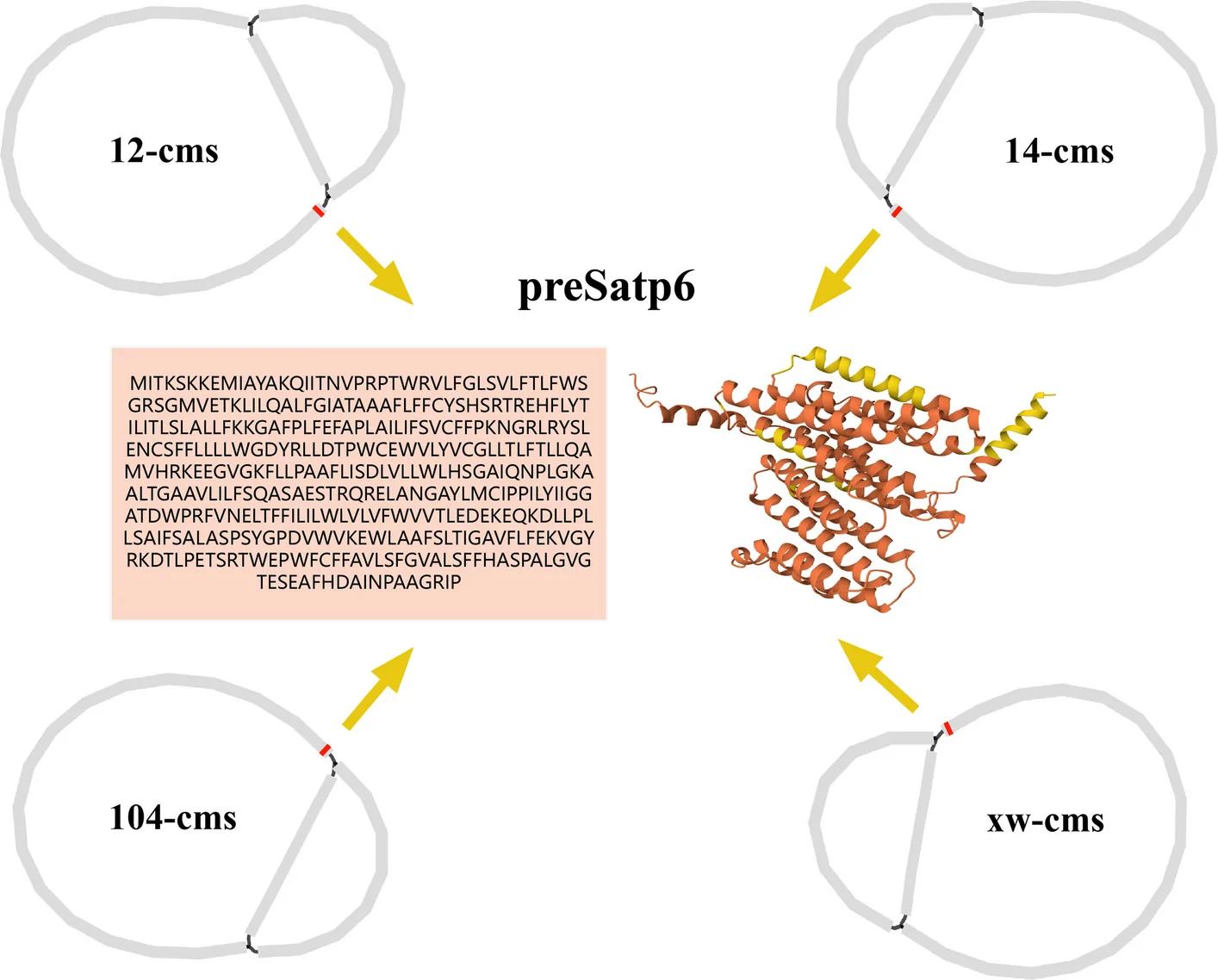
Identification of the *preSatp6* sequence in sugar beet CMS germplasm. Colored regions represent the *preSatp6* sequence. The orange box shows the deduced amino acid sequence of *preSatp6*, and the right panel shows its predicted protein structure

All functional genes in the beet mitochondrial genomes were annotated using the PMGA online platform combined with manual curation. A core set of 53 genes was conserved across all accessions, including 31 protein-coding genes or coding DNA sequences (CDSs), three ribosomal RNA (rRNA) genes, and 19 transfer RNA (tRNA) genes (Supplementary Fig. S3). These genes were categorized into 12 functional groups, such as ATP synthase subunits, cytochrome c biogenesis factors, and ubiquinol cytochrome c reductase components (Supplementary Table S1). After simplifying the genome configurations, each sample contained three contigs. Annotation of a representative sterile line (104-cms) is shown in Fig. 2. The Owen-type CMS-associated sequence *preSatp6* [12], a previously reported CMS-specific gene in sugar beet, was identified by BLASTN searches against the assembled mitochondrial contigs (see Materials and methods for detailed parameters) and found exclusively in the four CMS lines that were independently confirmed as sterile by breeding records (Fig. 3).The *preSatp6* sequence is located upstream of the *atp6* gene and contains a 387-amino-acid open reading frame (ORF). The predicted preSATP6 protein contains a putative N-terminal transmembrane domain (residues 8–30), suggesting mitochondrial membrane localisation, consistent with previous reports [12]. The protein is predicted to form a helical structure with a hydrophobic core, which may facilitate its integration into the mitochondrial inner membrane and subsequent formation of the 200 kDa homomeric complex characteristic of Owen-type CMS [12].

### Genomic landscape of repetitive sequences

**Fig. 4:**
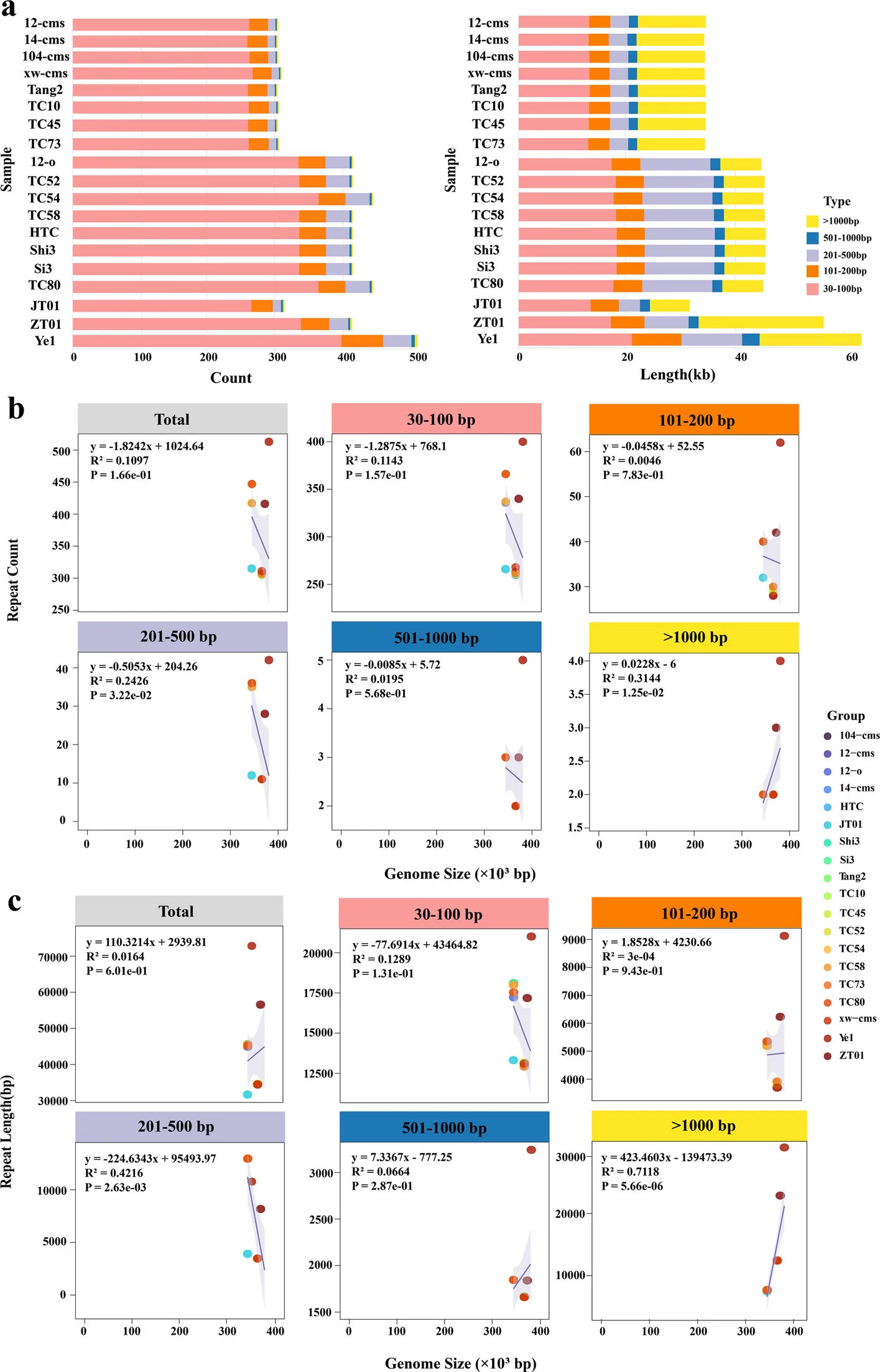
Characterization of repetitive sequences in 19 beet mitochondrial genomes. **a** Total count and cumulative length of repeats across five length categories. **b** Linear regression of repeat count per category against mitochondrial genome size (kb). **c** Linear regression of repeat length (bp) per category against mitochondrial genome size (kb). Each subplot in **b** and **c** displays the linear regression equation, coefficient of determination (R^2^), and two-tailed Student's t-test *P*-value

Repetitive sequences in the assembled genomes were divided into five length categories: 30–100 bp, 101–200 bp, 201–500 bp, 501–1000 bp, and > 1000 bp. Their abundance and cumulative length were quantified across all accessions (Fig. 4a; Supplementary Tables S3–S4). Quantitatively, short repeats dominated numerically: the 30–100 bp category contained 5,859 copies, whereas the 501–1000 bp and > 1000 bp categories had only 51 and 41 copies, respectively (Fig. 4a; Supplementary Table S3). At the intra-repeat population level, the count of individual repeat fragments was significantly negatively correlated with fragment length only for repeats > 1000 bp (R = − 0.41, P < 0.001; Table S3). Thus, the beet mitochondrial genome is enriched in short repeats, and no universal linear relationship exists between repeat abundance and single fragment length across all categories.

In terms of cumulative length contribution, long repeats played a disproportionate role in mitogenome expansion. Repeats > 1000 bp, though few in number, contributed 223,027 bp (27.73% of total cumulative repeat length), while the most abundant 30–100 bp repeats contributed 298,725 bp (37.14%), ranking second (Fig. 4a; Supplementary Table S4). To further investigate the relationship between repetitive sequence accumulation and mitochondrial genome expansion, we performed linear regression analyses of repeat count and repeat length against genome size across the 19 accessions (Fig. 4b, c). For repeat count versus genome size (Fig. 4b), the > 1000 bp category showed a significant positive correlation (R^2^ = 0.3144, P = 0.0125), whereas the 201–500 bp category exhibited a significant negative correlation (R^2^ = 0.2426, P = 0.0322). No significant correlations were detected for total repeat count or for the other length categories (P > 0.05). For repeat length versus genome size (Fig. 4c), the > 1000 bp category again showed the strongest positive correlation (R^2^ = 0.7118, P = 5.66 × 10⁻⁶), confirming that extra-long repeats are the primary drivers of mitochondrial genome expansion. The 201–500 bp category displayed a significant negative correlation with genome size (R^2^ = 0.4216, P = 0.0026), while total repeat length and all other categories showed no significant associations (P > 0.05). Collectively, these regression analyses demonstrate that the accumulation of extra-long repeats (> 1000 bp) is the predominant factor driving mitochondrial genome size variation in beet, whereas the negative correlation observed for 201–500 bp repeats may reflect competitive displacement by longer repeats in expanded genomes.

Marked differences were observed between wild and cultivated beets. Wild beet exhibited higher overall repeat content and more active expansion of long fragments, with a total repeat length of 76,192 bp (20.02% of its genome), approximately 1.84‑fold higher than the cultivated average (41,296 bp, 10.89%). Both groups had the highest count in the 30–100 bp class, but wild beet exceeded cultivated accessions in the number, total length, and average length of > 1000 bp fragments.

In addition to dispersed repeats, simple sequence repeats (SSRs) were also analyzed. A total of 49–53 SSRs were identified across the 19 accessions, indicating limited variation in SSR abundance. Among the six repeat types (mono-, di-, tri-, tetra-, penta-, and hexanucleotide), mononucleotide repeats were the most abundant (39–42 loci per mitochondrial genome, 76.47–83.67% of total SSRs), followed by pentanucleotide repeats (7–8 loci). Trinucleotide repeats were consistently present with two copies across all samples (3.77–4.08% of total SSRs). Hexanucleotide repeats were least abundant (1–2 loci detected per mitochondrial genome) (Supplementary Table S5).

### Nucleotide substitution rates in organellar genomes

Nucleotide substitution rates were estimated for conserved protein-coding genes (PCGs) from 19 beet accessions and one outgroup (*Amaranthus tricolor*), representing 20 genomes in total. Analyses covered 79 plastid genes and 30 mitochondrial genes. For each gene, the synonymous substitution rate (*dS*, reflecting neutral evolution) and the non-synonymous substitution rate (*dN*, indicating selective pressure) were calculated. Intraspecific evolutionary divergence was quantified as root-to-tip branch lengths in phylogenetic trees reconstructed from *dN*/*dS* matrices (Supplementary Table S6).

**Fig. 5:**
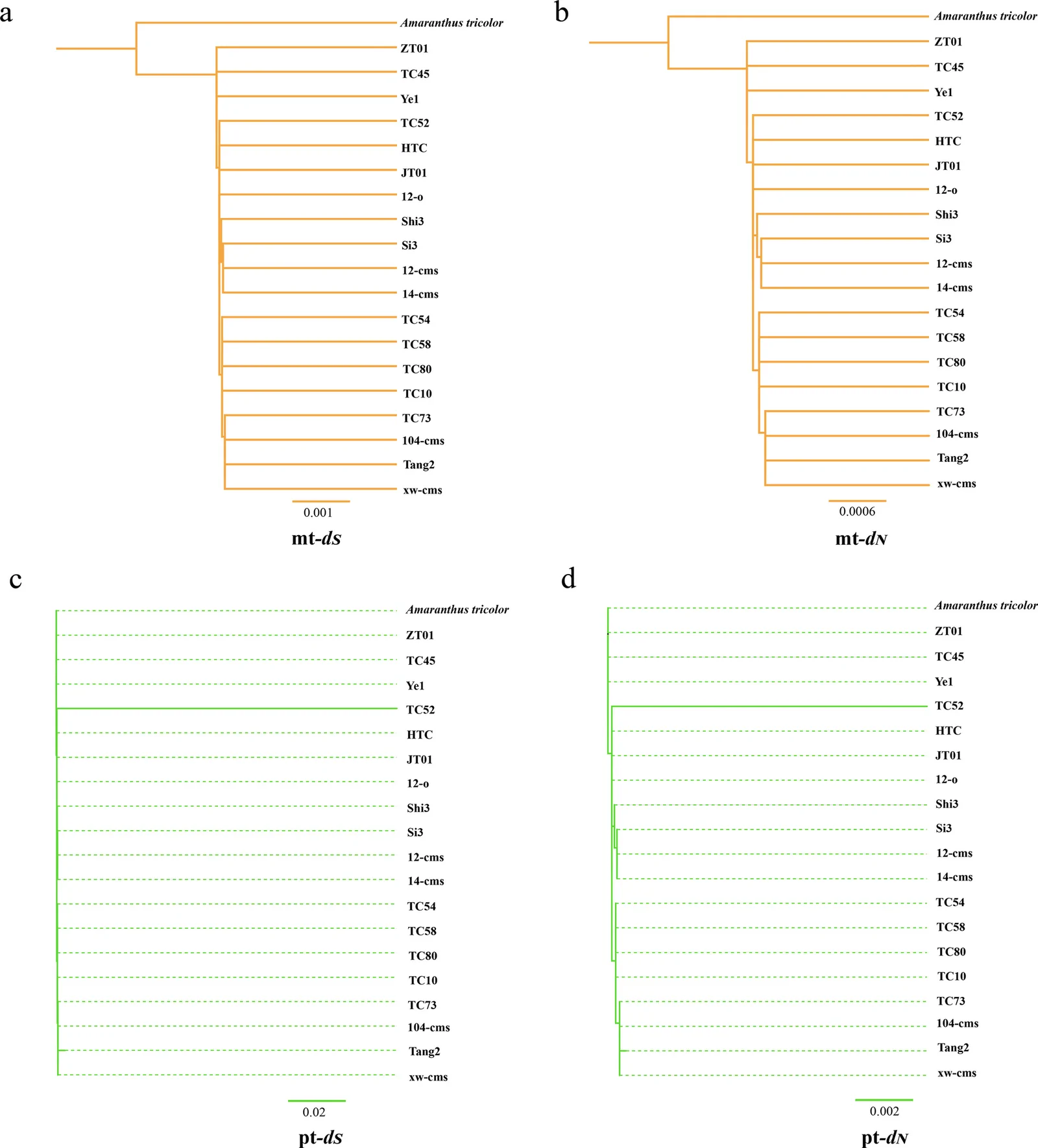
Visualization of cumulative nucleotide substitution rates in coding regions of beet organellar genomes. **a** Mitochondrial genome *dS* rate tree; **b** Mitochondrial genome *dN* rate tree; **c** Plastid genome *dS* rate tree; **d** Plastid genome *dN* rate tree. All four panels are displayed as phylograms with branch lengths proportional to the root‑to‑tip *dS/dN* values calculated by PAML codeml. Scale bars indicate substitutions per site. Absolute *dS/dN* values for all accessions are listed in Supplementary Table S6 for quantitative reference. Note that the plastid genome has a 10–100 fold lower substitution rate than the mitochondrial genome, resulting in shorter branch lengths in panels c and d

Phylogenetic analysis based on *dS* matrices revealed pronounced genomic type-specific differences in root-to-tip branch lengths between the mitochondrial and plastid genomes, with contrasting patterns of inter-accession variation. For the mitochondrial genome, root-to-tip *dS* ranged from 0.017377 to 0.018644, with the outgroup species *A. tricolor* exhibiting the lowest value within this interval. Among beet accessions, normal cytoplasmic lines displayed relatively low values with minor fluctuations, whereas CMS lines showed consistently elevated values with limited variation (Fig. 5a). CMS lines consistently showed elevated *dS* values (mean 0.0186) compared to non-CMS sugar beet accessions (mean 0.0176) (Table S6; Fig. 5a). In contrast, plastid *dS* values were substantially lower (0.0003–0.0049 for most accessions; several were 0), except for TC52, which displayed a notably high value of 0.1901 (Fig. 5c). This 10–100 fold magnitude difference in substitution rates between the mitochondrial and plastid genomes results in distinct visual presentations in Fig. 5: mitochondrial trees show more divergent branch lengths, while plastid trees appear visually compact. All branch relative lengths in Fig. 5 strictly correspond to the root-to-tip substitution values calculated by PAML codeml (full absolute values see Supplementary Table S6). Thus, the beet mitochondrial genome accumulated significantly more neutral substitutions than the plastid genome, and inter-accession differences in mitochondrial neutral evolution were more pronounced.

The *dN*-based trees showed similar patterns. Root-to-tip *dN* values were uniformly lower than the corresponding *dS* values across all accessions, indicating that amino-acid level variation accumulates more slowly than neutral substitutions. Mitochondrial *dN* ranged from 0.009 to 0.029 (longest in Tang2, shortest in Ye1) (Fig. 5b). Plastid *dN* ranged from 0 to 0.0247, with most accessions between 0.0003 and 0.0014; ZT01, Ye1, and *A. tricolor* had values of 0, and TC52 again showed an elevated value (0.0247) (Fig. 5d).

### Intracellular DNA transfer patterns in beet genomes

1. Plastid-to-mitochondrion DNA transfers (PTMTs)

**Fig. 6:**
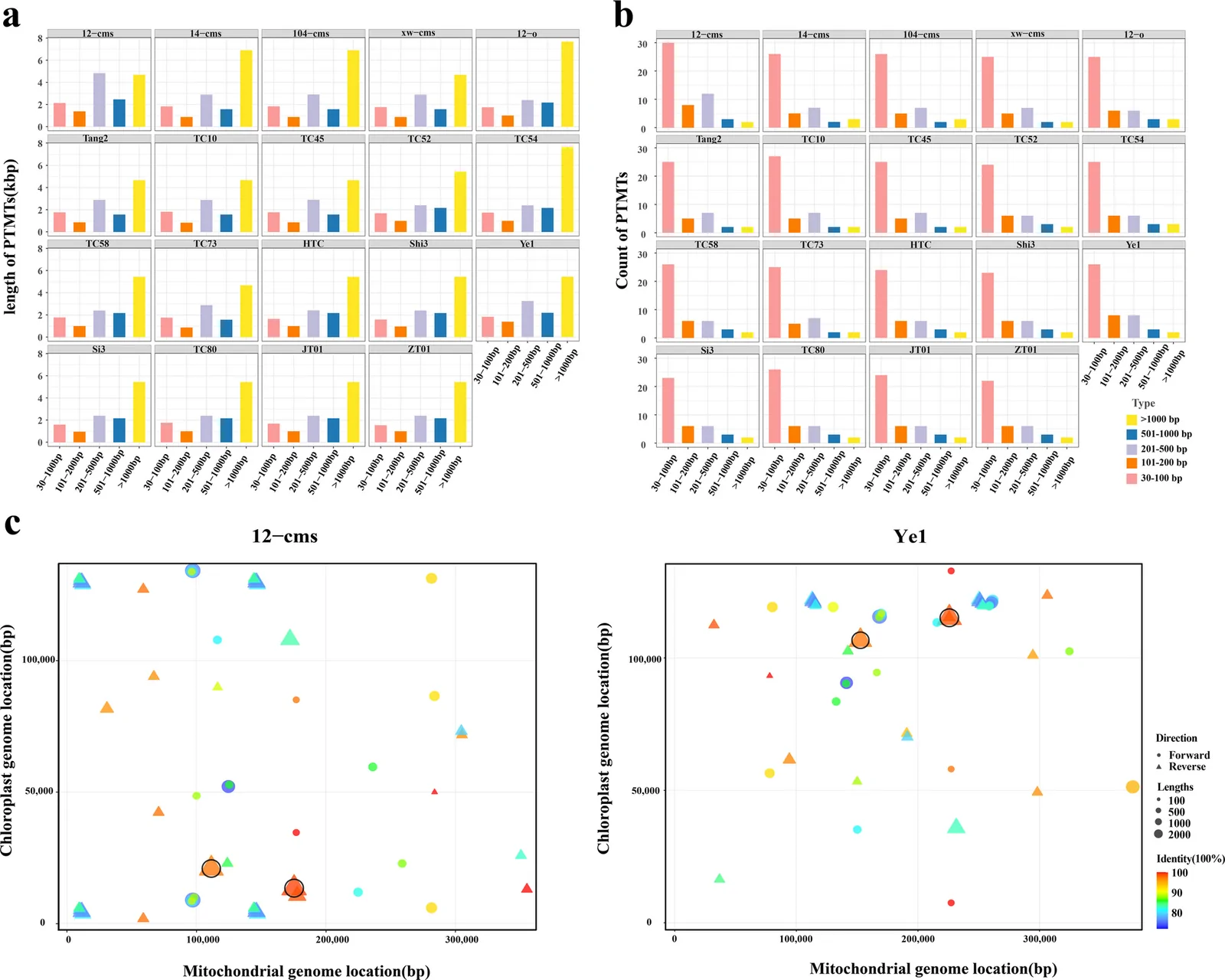
Characterization of PTMT fragments in beet genomes. **a** Length distribution; **b** Fragment count; **c** Genomic distribution of PTMT insertions in 12-cms and Ye1.Note for panel c: The x-axis is virtually concatenated from three assembled mitogenome contigs. Contig ranges on the axis:12-cms: cnt1 (0–221,477 bp), cnt2 (221,478–309,481 bp), cnt3 (309,482–365,891 bp); Ye1: cnt1 (0–133,290 bp), cnt2 (133,291–257,398 bp), cnt3 (257,399–380,704 bp). The y-axis indicates plastid genome positions; triangles/circles mark forward/reverse fragments; marker size = fragment length; color = sequence identity

A total of 809 plastid-derived fragments (PTMTs) were identified across the 19 beet mitochondrial genomes. Their abundance varied from 39 to 55 fragments per genome, and cumulative length from 11,773 to 15,481 bp (Fig. 6a, b). Short fragments (30–100 bp) were most numerous (477 fragments, 59.0% of total PTMTs) but contributed only 13.4% (33,312 bp) of total PTMT length. In contrast, long fragments (> 1000 bp) were rare (42 fragments) but accounted for 42.8% (106,211 bp) of total PTMT length.

Two representative accessions, 12-cms (CMS line) and Ye1 (wild beet), were selected to visualize the origins and genomic positions of PTMT insertions (Fig. 6c; Supplementary Fig. S4). To provide an intuitive holistic view of the genome-wide distribution pattern, the three assembled contigs of each mitogenome were concatenated into a virtual linear coordinate for plotting; contig IDs and their corresponding coordinate boundaries are clearly annotated in the figure legend.

In Ye1, PTMT insertions clustered predominantly within the first ~ 150 kb of the concatenated coordinate, corresponding to the entire cnt1 and the proximal segment of cnt2. In 12-cms, by contrast, PTMTs were distributed more uniformly across the 0–300 kb range of the concatenated coordinate, spanning cnt1, cnt2 and the proximal portion of cnt3. Multiple overlapping insertions were observed in several regions, suggesting the presence of integration hotspots for foreign DNA. Most PTMT fragments in both accessions shared > 80% sequence identity with their plastid donor sequences, and approximately one-third showed 100% identity.

(2) Nuclear organellar DNA insertions (NUMTs and NUPTs).

Nuclear mitochondrial DNA fragments (NUMTs) and nuclear plastid DNA fragments (NUPTs) were identified using the published beet nuclear genome EL10 as reference [24]. Across the 19 accessions, 18,654 NUMT fragments totaling 6,986,861 bp were found (Supplementary Fig. S5). Short NUMTs (< 200 bp) dominated numerically (79.8%) but contributed only 27.1% of total length, whereas long NUMTs (> 2000 bp) were rare (1.9%) but contributed 26.3% of total length. For NUPTs, 20,627 fragments totaling 7,628,769 bp were identified (Supplementary Fig. S5), with stable abundance across accessions (average 1,085 fragments; 401,514 bp). Short NUPTs (< 200 bp) were most numerous (59.7%) but contributed 30.4% of total length, while long NUPTs (> 2000 bp) were rare (2.7%) yet contributed 38.7% of total length. Thus, both NUMTs and NUPTs are characterized by numerical predominance of short fragments but substantial length contribution from long fragments. Per-accession raw statistics of NUMTs and NUPTs are fully listed in Supplementary Table S7.

(3) Age distribution and chromosomal localization.

**Fig. 7:**
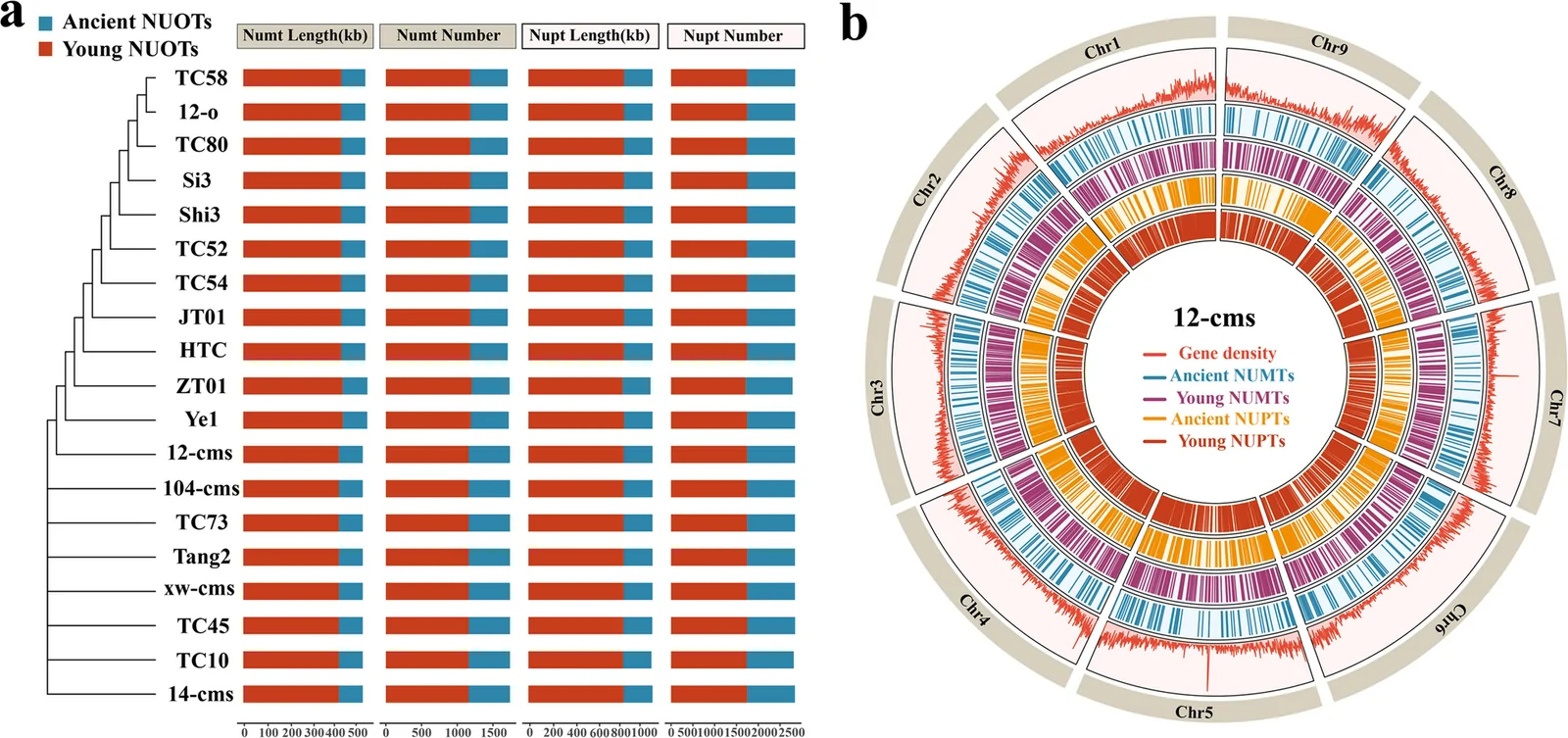
Distribution characteristics of NUMTs and NUPTs in the beet genome. **a** Genome-wide total number and cumulative length based on the phylogenetic tree of mitochondrial CDS sequences; **b** Circos plot showing the distribution of ancient and recent insertions across nuclear chromosomes of accession 12-cms

Based on sequence divergence, recent transfers substantially exceeded ancient ones in both length and number for NUMTs and NUPTs (Fig. 7a; Supplementary Table S7), supporting progressive degradation of organellar insertions in the nuclear genome over evolutionary time. Chromosomal localization revealed non-random insertion patterns for ancient NUMTs and NUPTs (Fig. 7b; Supplementary Fig. S6).

### Organellar genome phylogenies of beet accessions

**Fig. 8:**
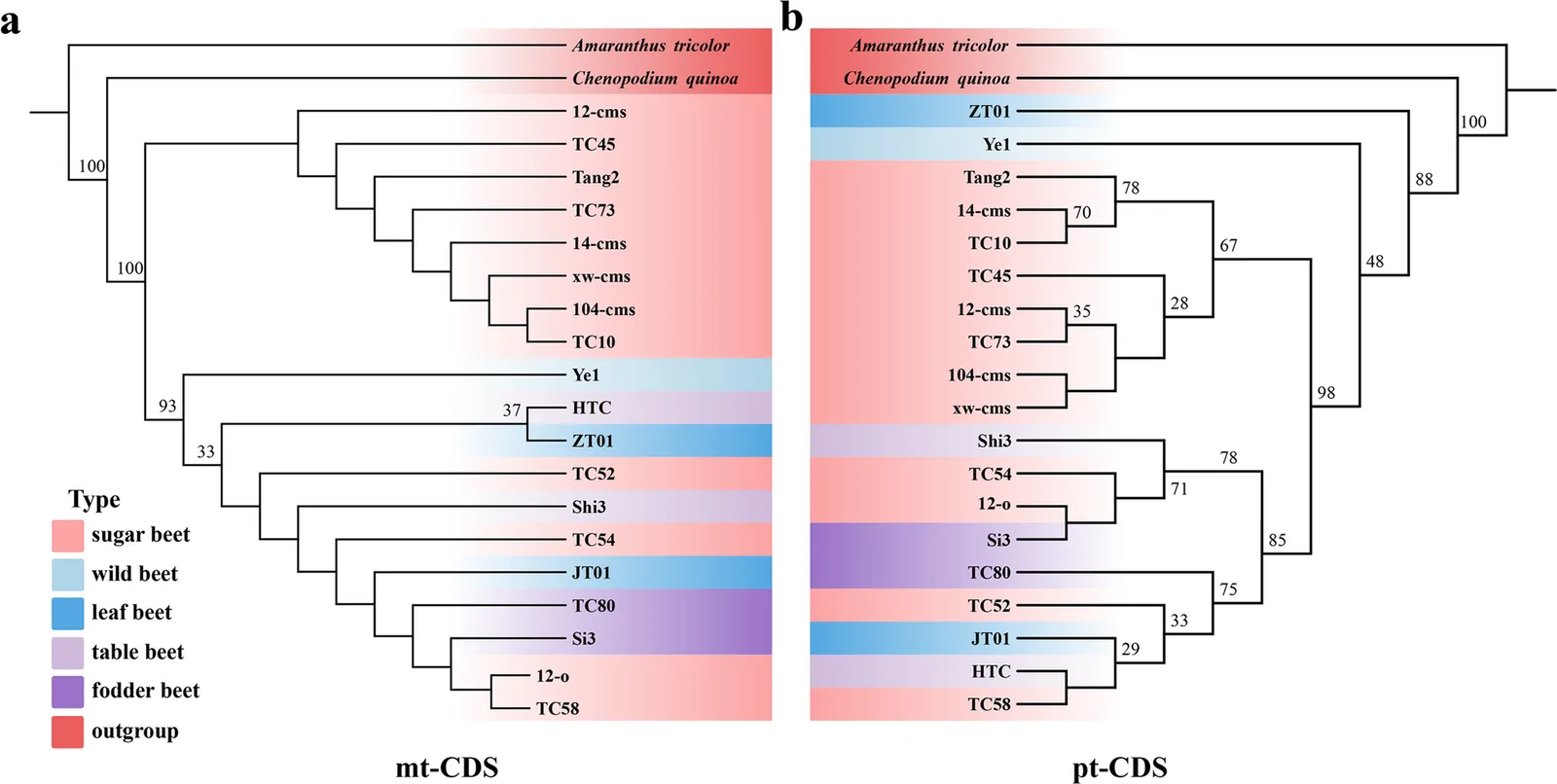
Phylogenetic relationships of beet. **a** Maximum likelihood (ML) tree based on concatenated CDSs from mitochondrial genomes; **b** ML tree based on concatenated CDSs from plastid genomes

Phylogenetic trees were constructed from 29 shared mitochondrial CDS and 78 shared plastid CDS of 21 accessions, with quinoa (*Chenopodium quinoa*) and amaranth (*Amaranthus tricolor*) as outgroups (Fig. 8). In both organellar trees, the outgroups formed independent clades clearly separated from all beet accessions. Wild beet (Ye1) occupied a basal position in the plastid tree (Fig. 8b) but was placed within cultivated beet clades in the mitochondrial tree (Fig. 8a), with generally low bootstrap support for deep nodes. Within cultivated beets, eight accessions (including four CMS lines and four non-CMS sugar beet accessions) formed a moderately supported clade (bootstrap = 87%) in the mitochondrial tree and a well‑supported clade (bootstrap = 100%) in the plastid tree, indicating a shared cytoplasmic ancestry among these accessions. Among these, the four CMS lines (12‑cms, 14‑cms, 104‑cms, xw‑cms) carried the *preSatp6* sequence, whereas the other four accessions (TC45, Tang2, TC73, TC10) lacked this marker.

However, the two trees showed notable discordance for non-CMS accessions. In the mitochondrial tree, leaf beets ZT01 and JT01 clustered with some fodder and table beets, whereas in the plastid tree, leaf beets were closer to the main sugar beet group. Table beets HTC and Shi3, together with fodder beets Si3 and TC80, resolved in different clades in the plastid tree. Accessions 12-o, JT01, and HTC formed a relatively independent evolutionary clade in the mitochondrial tree, indicating close mitochondrial genetic relationships. CMS lines (e.g., 12-cms, 14-cms) showed consistent high clustering in both trees, though clade positions and support values differed. Some secondary clades in the mitochondrial tree had low support (e.g., between non-CMS sugar beets and leaf beets), whereas the plastid tree exhibited higher support for key clades including CMS lines, outgroups, and cultivated types.

## Discussion

### Coexistence of evolutionary conservation and dynamic variation in mitochondrial genomes

Our assemblies of 19 beet accessions, covering diverse cultivated types (including CMS lines) and a wild representative, revealed both conserved and dynamic features of the beet mitochondrial genome. Genome sizes (344–381 kb) were consistent with previous reports [31]. Notably, the mitochondrial genomes formed three structural types based on the number of repetitive contigs: three in maintainer lines, two in CMS lines, and three in wild beet. This structural polymorphism likely reflects distinct recombination histories. The reduction from three to two contigs in CMS lines is correlated with the Owen-type CMS-inducing rearrangement, as repetitive sequences are known to mediate recombination events that generate chimeric CMS-associated ORFs [8]. However, whether this structural reduction directly results from increased recombination activity in CMS mitochondria, or reflects a historical fixation of a specific recombination product during CMS selection, cannot be determined from our data. The three-contig configuration in wild beet, shared with maintainer lines, suggests an ancestral state.

Wild beet exhibited a substantially larger genome (380,704 bp) and higher repeat content (1.84-fold the cultivated average), particularly in extra-long repeats. These features suggest that wild beet may retain a greater potential for structural variation under natural selection, although direct assays of recombination activity or isoform turnover would be required to confirm this hypothesis, whereas cultivated beet has become structurally simplified and stabilized under artificial directional selection. Mower et al. also observed rapid turnover of large repeats in Digitalideae mitochondrial genomes, consistent with a full turnover model [32]. Domestication thus appears to have reduced recombinationally active repeats, streamlining the mitochondrial genome in cultivated beets.

In contrast to structural dynamism, functional conservation was striking. All accessions shared the same 53 core genes, including intact energy-metabolism genes, with no gain or loss between wild and cultivated beets. This indicates that core mitochondrial functions have remained intact throughout domestication, unaffected by cultivar or cytoplasmic type. Minimal GC content variation further supports base composition stability. Together, these findings align with the general rule in higher plant mitochondrial genomes: despite frequent structural rearrangements, core gene content and function are highly conserved [33]. The duality of functional stability and structural dynamism in beet mitochondrial genomes provides a genomic backdrop for CMS evolution, where repeat-mediated rearrangements can drive agronomically relevant changes without compromising essential functions.

### Specificity and evolutionary significance of CMS-associated sequences

The *preSatp6* sequence was identified in the four CMS lines (which were independently confirmed as sterile by breeding records) and was absent in all non-CMS accessions, including the four non-CMS sugar beet accessions that cluster with them phylogenetically. This finding validates the diagnostic value of *preSatp6* as a marker for Owen-type CMS cytoplasm [12]. This chimeric sequence upstream of *atp6* is thought to disrupt pollen development by interfering with *atp6* expression or function [4]. Nucleotide substitution analysis revealed a clear gradient: wild beet exhibited the lowest *dS* and *dN* values, normal cytoplasmic cultivated lines had intermediate values, and CMS lines showed the highest values. The elevated substitution rates in CMS lines (mean *dS* = 0.0186) relative to non-CMS sugar beet accessions (mean *dS* = 0.0176) suggest accelerated evolution associated with the CMS cytoplasmic state. This pattern suggests that natural selection maintains a conservative evolutionary rate in wild beet, whereas artificial selection and altered reproductive modes (e.g., clonal propagation of CMS as female parents) have accelerated evolution in cultivated beets, especially in CMS lines [15].

The *dN*/*dS* ratios of both organellar genomes were far below 1, but mitochondrial genome ratios were consistently higher than plastid ratios, indicating that mitochondrial protein-coding genes experience weaker purifying selection than plastid genes, a pattern generally observed in plants [1]. Consequently, mitochondrial genes may harbor more adaptive variation related to fertility. Root-to-tip branch lengths further supported these genome-specific patterns: mitochondrial branch lengths were highest and stable in CMS lines, intermediate in normal cytoplasm lines, and shortest in wild beet, whereas plastid branch lengths showed no clustering by cytoplasm type, implying that plastid evolution is largely decoupled from cytoplasmic background. Mutations in plant mtDNA likely result from the interaction of multiple factors rather than any single dominant one [26]. Collectively, these results demonstrate that the Owen-type CMS mitochondrial genome evolves faster under relaxed purifying selection. The elevated substitution rates in CMS lines are likely attributable to a combination of factors, including reduced effective population size due to clonal propagation, possible recombination-associated mutagenesis, and relaxed selection resulting from the decoupling of male fertility from fitness in CMS breeding programs. However, as we did not directly quantify recombination rates or mutation spectra in this study, the relative contributions of these factors remain to be determined in future work, as well as the long-term “sheltered” transmission of CMS cytoplasm in breeding [27]. These findings provide evolutionary insights into CMS dynamics and underscore the need to monitor mitochondrial genome stability in CMS breeding materials.

### Universality and line-specificity of intracellular DNA transfer

A total of 809 PTMTs were identified across the 19 beet mitochondrial genomes, with an aggregate length of 247,962 bp. Short fragments dominated numerically but contributed little to total length, whereas long fragments were rare but accounted for most of the inserted sequence. This pattern suggests that plastid DNA integration into mitochondria occurs mainly through frequent infiltration of small fragments, while long-fragment retention requires specific structural conditions or selective advantages. PTMT abundance, total length, and genomic distribution varied markedly among accessions, with integration clustering in line-specific hotspot regions, likely constrained by local repeat distribution and recombination activity [34]. Approximately one-third of PTMTs showed 100% identity to their plastid sources, indicating either recent transfer events or strong purifying selection after integration.

Frequent transfer of organellar DNA to the nucleus was also evident from the widespread presence of NUMTs and NUPTs. Based on alignment against the unified EL10 nuclear reference, CMS lines carried a higher number of NUMT fragments (mean 1,739 per accession) than non-CMS sugar beet accessions (mean 1,707 per accession), while the mean total NUMT length was slightly lower in CMS lines (542,214 bp) than in non-CMS lines (553,701 bp) (Supplementary Table S7). This pattern is preliminarily consistent with a hypothesis that CMS mitochondrial genomes with active structural rearrangements may release more small DNA fragments available for nuclear integration.

However, two critical constraints prevent definitive conclusions. First, direct measurements of mitochondrial recombination rates, recombination hotspot activity or structural isoform abundance were not performed in this study; the association between NUMT accumulation and mitochondrial recombination remains purely correlative. Alternative explanations, such as differential nuclear integration efficiencies, selection on nuclear-localized organellar fragments, or specific cytonuclear genotype interactions, cannot be excluded [35, 36]. Second, a methodological caveat applies to all cross-accession NUMT/NUPT comparisons: our analysis used a single sugar beet nuclear genome (EL10) as the common reference, so detected insertions only reflect fragments homologous to the EL10 background and cannot capture accession-specific nuclear structural variation. The observed NUMT differences between CMS and non-CMS lines may partly stem from organellar genome sequence divergence relative to EL10, rather than true differences in nuclear insertion abundance among germplasms.

Taken together, the potential link between NUMT accumulation and CMS cytoplasmic status is proposed as a testable hypothesis rather than a validated conclusion. Definitive verification of functional cytonuclear interactions will require chromosome-scale de novo nuclear genome assemblies for each studied accession to eliminate reference bias in future work. In contrast, NUPTs were highly consistent across all lines, implying tighter nuclear regulation of plastid DNA integration. Age distribution analysis showed that recent transfers (both NUMTs and NUPTs) substantially outnumbered ancient ones in length and count, supporting progressive degradation of organellar insertions in the nuclear genome over evolutionary time [37, 38]. Such insertions gradually lose original characteristics through base mutations, recombination, and deletions, a universal evolutionary pattern of heterologous DNA in nuclear genomes [25].

### Phylogenetic relationships and domestication history of cultivated and wild beet

Phylogenetic trees based on plastid and mitochondrial CDS revealed both congruent and discordant signals regarding beet domestication. Wild beet (Ye1) occupied a basal position in the plastid tree, consistent with its role as the ancestral cytoplasmic donor. However, in the mitochondrial tree, wild beet did not clearly separate from cultivated beets, and deep nodes received low bootstrap support, suggesting that mitochondrial genome evolution in beet has been influenced by recombination and structural rearrangements that obscure phylogenetic signals at deeper levels [41]. Within cultivated beets, eight accessions formed a well-supported clade in the plastid tree (bootstrap = 100%) and a moderately supported clade in the mitochondrial tree (bootstrap = 87%), consistent with a common maternal origin shaped by long-term directional introgression and strong selection.

The two organellar trees showed notable discordance in the clustering of non-CMS sugar beet, leaf beet, fodder beet, and table beet. This incongruence is a common phenomenon in plants, arising from differences in maternal inheritance, high mitochondrial recombination rates, and nucleo-cytoplasmic selection [39]. Plastids follow strict maternal inheritance and thus preserve domestication signals more stably, offering stronger resolution for major group differentiation [40]. Mitochondria, in contrast, are more susceptible to recombination, CMS-related fragment insertions, backcross introgression, and artificial selection, leading to more complex evolutionary trajectories, lower branch support in some clades, and topological discordance with plastid trees [41]. These differences underscore that beet domestication involved co-evolution between nuclear genes and cytoplasmic genomes, with wild germplasm providing the original source of cytoplasmic diversity for cultivated beets.

## Conclusions

Beet mitochondrial genomes exhibit a fundamental duality. Core functional genes remain highly conserved across all cultivated types and wild beet, ensuring essential respiratory functions. In contrast, structural architecture varies markedly under different selection pressures. Wild beet represents the ancestral state by retaining a larger repeat-rich genome with slower evolutionary rates. Domestication and artificial selection have streamlined mitochondrial genome structure in cultivated beets. CMS lines show a distinctive two-contig configuration, significantly elevated substitution rates, and the exclusive presence of the *preSatp6* sequence. These features are associated with the CMS phenotype, although the mechanistic relationships between structural configuration, substitution rates, and sterility require further experimental validation, a derived trait absent in the wild progenitor. These CMS-associated genomic features are further reflected in intracellular DNA transfer patterns. Plastid-to-mitochondrion transfers are widespread but lineage-specific, and nuclear mitochondrial insertions accumulate preferentially in CMS lines, indicating active cytonuclear interaction. Phylogenetically, all CMS lines share a single maternal origin, and wild beet occupies the basal position as the ancestral cytoplasmic donor. Collectively, these findings establish that beet domestication has driven structural simplification and accelerated mitochondrial genome evolution in CMS lines while preserving core functional integrity. This work provides a genomic framework for CMS marker development and cytoplasmic breeding.

## Supplementary Information

Supplementary Material 1: Fig. S1. Circular map of the beet mitochondrial genome. Genes located outside the outer circle are transcribed in the forward direction, whereas those inside the outer circle are transcribed in reverse direction. The carbon-grey inner circle indicates GC content, with distinct color shades representing different functional gene groups. Fig. S2. Assembly maps of beet mitochondrial genomes. (a) Configuration with one repetitive contig; (b) Configuration with two repetitive contigs; (c) Configuration with three repetitive contigs. Different colors in the maps represent sequence homology between assembled fragments and the reference genome sequence; identical colors indicate regions of sequence homology. Fig. S3. Annotated genes in the beet mitochondrial genome. Fig. S4. Comparative analysis of the origin positions and insertion sites of PTMT fragments. The horizontal and vertical axes represent positions (bp) in the mitochondrial and plastid genomes, respectively. Triangles and circles indicate forward and reverse fragments, respectively. Marker size corresponds to fragment length, and color reflecting sequence identity. Fig. S5. Characterization of nuclear organellar DNA transfer fragments in the beet genome. (a) Length distribution of NUMT fragments; (b) Number distribution of NUMT fragments; (c) Length distribution of NUPT fragments; (d) Number distribution of NUPT fragments. Fig. S6. Distribution of ancient and young transferred NUMTs and NUPTs in the nuclear chromosomes of beet. Table S1. List of beet accessions and their cytoplasmic characteristics. Table S2. Annotated genes in the mitochondrial genome of beet. Table S3. Summary of repeat sequence counts in the beet mitochondrial genome. Table S4. Summary of repeat sequence lengths in the beet mitochondrial genome. Table S5. Quantity distribution of different simple sequence repeat (SSR) types in the beet mitochondrial genome. Table S6. Estimation of nucleotide substitution rates in beet organelle genomes. Table S7. Detection of nuclear plastid DNA (NUPT) and nuclear mitochondrial DNA (NUMT).

## Data Availability

The datasets generated and analyzed in this article are available in the Figshare repository (https://figshare.com/) with DOI: https://doi.org/10.6084/m9.figshare.32297004. All other data and material analyzed in the current study are included in the manuscript and the supplementary information.

## Abbreviations

CMS: Cytoplasmic male sterility
PTMTs: Plastid‑to‑mitochondrion transfers
NUPTs: Nuclear plastid DNA fragments
NUMTs: Nuclear mitochondrial DNA fragments
ML: Maximum likelihood
GTR: General time‑reversible
CDSs: Coding DNA sequences
rRNA: Ribosomal RNA
tRNA: Transfer RNA
SSRs: Simple sequence repeats
PCCs: Protein-coding genes
ORFs: Open reading frames
