# Supplementary material for: Mitogenomic variation across cultivated and wild beet (Beta vulgaris L.) accessions reveals patterns associated with cytoplasmic male sterility
Source: BMC Plant Biol. 2026 Aug 17;26:1937. doi: 10.1186/s12870-026-09766-2 (PMC13756948; doi:10.1186/s12870-026-09766-2)
Supplement: Supplementary file 1 [file 12870_2026_9766_MOESM1_ESM.zip › Table S7. Detection of nuclear plastid DNA (NUPT) and nuclear mitochondrial DNA (NUMT)..docx]

**Table S7** Detection of nuclear plastid DNA (NUPT) and nuclear mitochondrial DNA (NUMT).

| **Sample** | **Ancient and young NUMT** | | **Ancient and young NUPT** | |
| --- | --- | --- | --- | --- |
|  | **Length (bp)** | **number** | **Length (bp)** | **number** |
| 12-cms | 108,353/433,861 | 578/1,161 | 253,978/838,858 | 1,097/1,745 |
| 14-cms | 108,353/433,861 | 578/1,161 | 253,728/838,852 | 1,096/1,745 |
| 104-cms | 108,353/433,861 | 578/1,161 | 253,744/838,847 | 1,096/1,745 |
| xw-cms | 108,353/433,861 | 578/1,161 | 254,018/838,882 | 1,096/1,745 |
| 12-o | 108,883/444,818 | 523/1,184 | 251,324/841,635 | 1,099/1,746 |
| Tang2 | 108,353/433,861 | 578/1,161 | 254,015/838,874 | 1,096/1,745 |
| TC10 | 108,353/433,861 | 578/1,161 | 250,669/832,248 | 1,083/1,730 |
| TC45 | 108,353/433,861 | 578/1,161 | 253,721/836,816 | 1,095/1,748 |
| TC52 | 108,883/444,818 | 523/1,184 | 251,618/841,823 | 1,110/1,746 |
| TC54 | 108,883/444,818 | 523/1,184 | 251,200/841,811 | 1,097/1,747 |
| TC58 | 108,883/444,818 | 523/1,184 | 251,615/841,668 | 1,099/1,746 |
| TC73 | 108,353/433,861 | 578/1,161 | 254,019/838,889 | 1,096/1,745 |
| HTC | 108,883/444,818 | 523/1,184 | 251,452/842,039 | 1,099/1,747 |
| Shi3 | 108,883/444,818 | 523/1,184 | 251,724/841,807 | 1,099/1/747 |
| Ye1 | 110,281/452,117 | 530/1,191 | 251,330/841,756 | 1,099/1,745 |
| Si3 | 108,883/444,818 | 523/1,184 | 251,663/841,268 | 1,099/1,748 |
| TC80 | 108,883/444,818 | 523/1,184 | 251,333/840,992 | 1,097,1,744 |
| JT01 | 108,883/444,818 | 523/1,184 | 251,607/841,661 | 1,099/1,746 |
| ZT01 | 112,378/450,769 | 531/1,202 | 246,234/829,013 | 1,073/1,713 |
