# Supplementary material for: Mitogenomic variation across cultivated and wild beet (Beta vulgaris L.) accessions reveals patterns associated with cytoplasmic male sterility
Source: BMC Plant Biol. 2026 Aug 17;26:1937. doi: 10.1186/s12870-026-09766-2 (PMC13756948; doi:10.1186/s12870-026-09766-2)
Supplement: Supplementary file 1 [file 12870_2026_9766_MOESM1_ESM.zip › Table S4 Summary of the lengths of repeat sequences in the beet mitochondrial genome.docx]

**Table S4** Summary of repeat sequence lengths in the beet mitochondrial genome.

| **Sample** | **Genome size (bp)** | **Total repeat length (bp)** | **Length 30–100 bp** | **Length 101–200 bp** | **Length 201–500 bp** | **Length 501–1,000 bp** | **Length >1,000 bp** |
| --- | --- | --- | --- | --- | --- | --- | --- |
| 12-cms | 365,891 | 34,491 | 13,111 | 3,701 | 3,469 | 1,662 | 12,548 |
| 14-cms | 365,891 | 34,645 | 13,065 | 3,905 | 3,462 | 1,662 | 12,551 |
| 104-cms | 365,891 | 34,446 | 13,074 | 3,697 | 3,465 | 1,662 | 12,548 |
| xw-cms | 365,891 | 34,491 | 13,111 | 3,701 | 3,469 | 1,662 | 12,548 |
| 12-o | 345,008 | 44,903 | 17,224 | 5,311 | 12,953 | 1,846 | 7,569 |
| Tang2 | 365,891 | 34,637 | 13,169 | 3,790 | 3,463 | 1,664 | 12,551 |
| TC10 | 365,892 | 34,620 | 13,029 | 3,905 | 3,462 | 1,673 | 12,551 |
| TC45 | 365,894 | 34,637 | 13,169 | 3,790 | 3,463 | 1,664 | 12,551 |
| TC52 | 345,008 | 45,544 | 17,995 | 5,185 | 12,949 | 1,846 | 7,569 |
| TC54 | 345,008 | 45,247 | 17,530 | 5,353 | 12,949 | 1,846 | 7,569 |
| TC58 | 345,008 | 45,544 | 17,995 | 5,185 | 12,949 | 1,846 | 7,569 |
| TC73 | 365,891 | 34,509 | 12,914 | 3,919 | 3,463 | 1,662 | 12,551 |
| HTC | 345,008 | 45,670 | 18,117 | 5,188 | 12,950 | 1,846 | 7,569 |
| Shi3 | 345,009 | 45,630 | 18,071 | 5,191 | 12,953 | 1,846 | 7,569 |
| Ye1 | 380,704 | 76,192 | 21,008 | 9,127 | 11,232 | 3,242 | 31,583 |
| Si3 | 345,008 | 45,670 | 18,117 | 5,188 | 12,950 | 1,846 | 7,569 |
| TC80 | 345,008 | 45,247 | 17,530 | 5,353 | 12,949 | 1,846 | 7,569 |
| JT01 | 344,560 | 31,655 | 13,316 | 5,233 | 3,913 | 1,848 | 7,345 |
| ZT01 | 372,169 | 56,568 | 17,180 | 6,231 | 8,169 | 1,840 | 23,148 |
