# Supplementary material for: Mitogenomic variation across cultivated and wild beet (Beta vulgaris L.) accessions reveals patterns associated with cytoplasmic male sterility
Source: BMC Plant Biol. 2026 Aug 17;26:1937. doi: 10.1186/s12870-026-09766-2 (PMC13756948; doi:10.1186/s12870-026-09766-2)
Supplement: Supplementary file 1 [file 12870_2026_9766_MOESM1_ESM.zip › Table S3 Summary of the number of repeat sequences in the beet mitochondrial genome.docx]

**Table S3** Summary of repeat sequence counts in the beet mitochondrial genome.

| **Sample** | **Chr_len (bp)** | **Count 30–100 bp** | **Count 101–200 bp** | **Count 201–500 bp** | **Count 501–1,000 bp** | **Count >1,000 bp** | **Total count** |
| --- | --- | --- | --- | --- | --- | --- | --- |
| 12-cms | 365,891 | 263 | 28 | 11 | 2 | 2 | 306 |
| 14-cms | 365,891 | 260 | 30 | 11 | 2 | 2 | 305 |
| 104-cms | 365,891 | 263 | 28 | 11 | 2 | 2 | 306 |
| xw-cms | 365,891 | 268 | 28 | 11 | 2 | 2 | 311 |
| 12-o | 345,008 | 336 | 40 | 36 | 3 | 2 | 417 |
| Tang2 | 365,891 | 261 | 29 | 11 | 2 | 2 | 305 |
| TC10 | 365,892 | 262 | 30 | 11 | 2 | 2 | 307 |
| TC45 | 365,894 | 261 | 29 | 11 | 2 | 2 | 305 |
| TC52 | 345,008 | 337 | 40 | 35 | 3 | 2 | 417 |
| TC54 | 345,008 | 366 | 40 | 36 | 3 | 2 | 447 |
| TC58 | 345,008 | 337 | 40 | 35 | 3 | 2 | 417 |
| TC73 | 365,891 | 262 | 30 | 11 | 2 | 2 | 307 |
| HTC | 345,008 | 337 | 40 | 35 | 3 | 2 | 417 |
| Shi3 | 345,009 | 337 | 40 | 35 | 3 | 2 | 417 |
| Ye1 | 380,704 | 400 | 62 | 42 | 5 | 4 | 513 |
| Si3 | 345,008 | 337 | 40 | 35 | 3 | 2 | 417 |
| TC80 | 345,008 | 366 | 40 | 36 | 3 | 2 | 447 |
| JT01 | 344,560 | 266 | 32 | 12 | 3 | 2 | 315 |
| ZT01 | 372,169 | 340 | 42 | 28 | 3 | 3 | 416 |
