# Supplementary material for: Mitogenomic variation across cultivated and wild beet (Beta vulgaris L.) accessions reveals patterns associated with cytoplasmic male sterility
Source: BMC Plant Biol. 2026 Aug 17;26:1937. doi: 10.1186/s12870-026-09766-2 (PMC13756948; doi:10.1186/s12870-026-09766-2)
Supplement: Supplementary file 1 [file 12870_2026_9766_MOESM1_ESM.zip › Table S1. List of beet accessions and their cytoplasmic characteristics.docx]

**Table S1** List of beet accessions and their cytoplasmic characteristics.

| **Accession code** | **Cultivated type** | **Fertility/cytoplasm status** | ***preSatp6* presence** | **Source / notes** |
| --- | --- | --- | --- | --- |
| 12-cms | sugar beet | CMS | Yes | Owen‑type CMS line |
| 14-cms | sugar beet | CMS | Yes | Owen‑type CMS line |
| 104-cms | sugar beet | CMS | Yes | Owen‑type CMS line |
| xw-cms | sugar beet | CMS | Yes | Owen‑type CMS line |
| 12-o | sugar beet | maintainer (known fertile) | No | Maintainer for 12‑cms |
| Tang2 | sugar beet | non‑CMS (fertility not determined) | No | — |
| TC10 | sugar beet | non‑CMS (fertility not determined) | No | — |
| TC45 | sugar beet | non‑CMS (fertility not determined) | No | — |
| TC52 | sugar beet | non‑CMS (fertility not determined) | No | — |
| TC54 | sugar beet | non‑CMS (fertility not determined) | No | — |
| TC58 | sugar beet | non‑CMS (fertility not determined) | No | — |
| TC73 | sugar beet | non‑CMS (fertility not determined) | No | — |
| HTC | table beet | non‑CMS (fertility not determined) | No | — |
| Shi3 | table beet | non‑CMS (fertility not determined) | No | — |
| Ye1 | wild beet | wild (fertility not determined) | No | Wild progenitor |
| Si3 | fodder beet | non‑CMS (fertility not determined) | No | — |
| TC80 | fodder beet | non‑CMS (fertility not determined) | No | — |
| JT01 | leaf beet | non‑CMS (fertility not determined) | No | — |
| ZT01 | leaf beet | non‑CMS (fertility not determined) | No | — |
