# Supplementary material for: Mitogenomic variation across cultivated and wild beet (Beta vulgaris L.) accessions reveals patterns associated with cytoplasmic male sterility
Source: BMC Plant Biol. 2026 Aug 17;26:1937. doi: 10.1186/s12870-026-09766-2 (PMC13756948; doi:10.1186/s12870-026-09766-2)
Supplement: Supplementary file 1 [file 12870_2026_9766_MOESM1_ESM.zip › Table S2. Annotated genes in the mitochondrial genome of beet.docx]

**Table S2** Annotated genes in the mitochondrial genome of beet**.**

| **Gene group** | **Gene name** |
| --- | --- |
| ATP synthase | atp1, atp4, atp6, atp8, atp9 |
| Cytohrome c biogenesis | *ccmB,ccmC,ccmFC,ccmFN* |
| Ubichinol cytochrome c reductase | *cob* |
| Cytochrome c oxidase | *cox1,cox2,cox3* |
| Maturases | *matR* |
| Transport membrance protein | *mttB* |
| NADH dehydrogenase | *nad1,nad2,nad3,nad4,nad4L,nad5,nad6,nad7,nad9* |
| Ribosomal proteins (LSU) | *rpl5* |
| Ribosomal proteins (SSU) | *rps12,rps13,rps3,rps4,rps7* |
| Succinate dehydrogenase | *sdh4* |
| Ribosomal RNAs | rrn18,rrn26,rrn5 |
| Transfer RNAs | *trnE-UUC, trnS-GGA, trnS-GCU,trnS-UGA, trnY-GUA, trnM-CAU, trnD-GUA, trnD-GUC* |
|  | *trnI-CAU, trnK-UUU, trnC-GCA, trnN-GUU, trnP-UGG, trnF-GAA, trnH-GUG, trnW-CCA* |
|  | *trnG-GCC, trnQ-UUG, trnV-GAC* |
