# Supplementary material for: Mitogenomic variation across cultivated and wild beet (Beta vulgaris L.) accessions reveals patterns associated with cytoplasmic male sterility
Source: BMC Plant Biol. 2026 Aug 17;26:1937. doi: 10.1186/s12870-026-09766-2 (PMC13756948; doi:10.1186/s12870-026-09766-2)
Supplement: Supplementary file 1 [file 12870_2026_9766_MOESM1_ESM.zip › Table S5 Quantity distribution of different simple sequence repeat (SSR) types in the beet mitochondrial genome.docx]

**Table S5** Quantity distribution of different simple sequence repeat (SSR) types in the beet mitochondrial genome.

| **Sample** | **total** | **monomer** | **dimer** | **trimer** | **tetramer** | **pentamer** | **hexamer** |
| --- | --- | --- | --- | --- | --- | --- | --- |
| 12-cms | 51 | 39 | 0 | 2 | 0 | 8 | 2 |
| 14-cms | 49 | 39 | 0 | 2 | 0 | 7 | 1 |
| 104-cms | 49 | 39 | 0 | 2 | 0 | 7 | 1 |
| xw-cms | 49 | 39 | 0 | 2 | 0 | 7 | 1 |
| 12-o | 51 | 40 | 0 | 2 | 0 | 7 | 2 |
| Tang2 | 49 | 39 | 0 | 2 | 0 | 7 | 1 |
| TC10 | 49 | 39 | 0 | 2 | 0 | 7 | 1 |
| TC45 | 49 | 39 | 0 | 2 | 0 | 7 | 1 |
| TC52 | 51 | 40 | 0 | 2 | 0 | 7 | 2 |
| TC54 | 51 | 40 | 0 | 2 | 0 | 7 | 2 |
| TC58 | 51 | 40 | 0 | 2 | 0 | 7 | 2 |
| TC73 | 49 | 39 | 0 | 2 | 0 | 7 | 1 |
| HTC | 51 | 40 | 0 | 2 | 0 | 7 | 2 |
| Shi3 | 51 | 40 | 0 | 2 | 0 | 7 | 2 |
| Ye1 | 51 | 39 | 0 | 2 | 0 | 8 | 2 |
| Si3 | 51 | 40 | 0 | 2 | 0 | 7 | 2 |
| TC80 | 51 | 40 | 0 | 2 | 0 | 7 | 2 |
| JT01 | 51 | 40 | 0 | 2 | 0 | 7 | 2 |
| ZT01 | 53 | 42 | 0 | 2 | 0 | 7 | 2 |
