# Supplementary material for: Mitogenomic variation across cultivated and wild beet (Beta vulgaris L.) accessions reveals patterns associated with cytoplasmic male sterility
Source: BMC Plant Biol. 2026 Aug 17;26:1937. doi: 10.1186/s12870-026-09766-2 (PMC13756948; doi:10.1186/s12870-026-09766-2)
Supplement: Supplementary file 1 [file 12870_2026_9766_MOESM1_ESM.zip › Table S6 Estimation of nucleotide substitution rates in beet organelle genomes..docx]

**Table S6** Estimation of nucleotide substitution rates in beet organelle genomes.

| **Sample** | **Mitochondria** | | **plastid** | |
| --- | --- | --- | --- | --- |
|  | ***d*s** | ***d*n** | ***d*s** | ***d*n** |
| *Amaranthus tricolor* | 0.017377 | 0.007685 | 0.00000 | 0.00000 |
| 12-cms | 0.018614 | 0.008015 | 0.00070 | 0.00070 |
| 14-cms | 0.018624 | 0.008015 | 0.00070 | 0.00070 |
| 104-cms | 0.018644 | 0.008015 | 0.00090 | 0.00090 |
| xw-cms | 0.018634 | 0.008015 | 0.00100 | 0.00090 |
| 12-o | 0.017568 | 0.007804 | 0.00050 | 0.00030 |
| Tang2 | 0.018639 | 0.008015 | 0.00490 | 0.00140 |
| TC10 | 0.018644 | 0.008015 | 0.00060 | 0.00060 |
| TC45 | 0.018629 | 0.008015 | 0.00000 | 0.00000 |
| TC52 | 0.017538 | 0.007804 | 0.19010 | 0.02470 |
| TC54 | 0.017563 | 0.007804 | 0.00060 | 0.00060 |
| TC58 | 0.017548 | 0.007804 | 0.00070 | 0.00060 |
| TC73 | 0.018619 | 0.008015 | 0.00090 | 0.00090 |
| HTC | 0.017533 | 0.007804 | 0.00040 | 0.00030 |
| Shi3 | 0.017558 | 0.007804 | 0.00050 | 0.00050 |
| Ye1 | 0.017518 | 0.007854 | 0.00000 | 0.00000 |
| Si3 | 0.017553 | 0.007804 | 0.00070 | 0.00070 |
| TC80 | 0.017543 | 0.007804 | 0.00060 | 0.00060 |
| JT01 | 0.017568 | 0.007804 | 0.00030 | 0.00030 |
| ZT01 | 0.017523 | 0.007854 | 0.00000 | 0.00000 |
